# A novel synthetic synovial fluid model for investigating biofilm formation and antibiotic susceptibility in prosthetic joint infections

**DOI:** 10.1128/spectrum.01980-24

**Published:** 2024-11-29

**Authors:** Amber De Bleeckere, Frits van Charante, Thibault Debord, Stien Vandendriessche, Michiel De Cock, Marte Verstraete, Fabien Lamret, Bram Lories, Jerina Boelens, Fany Reffuveille, Hans P. Steenackers, Tom Coenye

**Affiliations:** 1 Laboratory of Pharmaceutical Microbiology, Ghent University, Ghent, Belgium; 2 Centre of Microbial and Plant Genetics (CMPG), Department of Microbial and Molecular Systems, KU Leuven, Leuven, Belgium; 3 Department of Medical Microbiology, Ghent University Hospital, Ghent, Belgium; 4 Université de Reims Champagne-Ardenne, BIOS, Reims, France; 5 Department of Diagnostic Sciences, Ghent University, Ghent, Belgium; 6 ESCMID Study Group on Biofilms (ESGB), Basel, Switzerland; Navarrabiomed-Universidad Pública de Navarra (UPNA)-Complejo Hospitalario de Navarra (CHN), IdiSNA, Pamplona, Navarra, Spain

**Keywords:** prosthetic joint infection, synovial fluid, biofilms

## Abstract

There is growing evidence that bacteria encountered in prosthetic joint infections (PJIs) form surface-attached biofilms on prostheses, as well as biofilm aggregates embedded in synovial fluid and tissues. However, *in vitro* models allowing the investigation of these biofilms and the assessment of their antimicrobial susceptibility in physiologically relevant conditions are currently lacking. To address this, we developed a synthetic synovial fluid (SSF2) model and validated this model by investigating growth, aggregate formation, and antimicrobial susceptibility using multiple PJI isolates belonging to various microorganisms. In this study, 18 PJI isolates were included belonging to *Staphylococcus aureus*, coagulase-negative staphylococci, *Cutibacterium acnes*, *Streptococcus* spp., *Enterococcus* spp., *Pseudomonas aeruginosa*, *Escherichia coli*, and *Candida* spp. Growth and aggregate formation in SSF2 were evaluated using light microscopy and confocal laser scanning microscopy. The biofilm preventing concentration (BPC) and minimal biofilm inhibitory concentration (MBIC) of relevant antibiotics were determined using a resazurin-based viability staining. BPC and MBIC values were compared to conventional susceptibility parameters (minimal inhibitory concentration and minimal bactericidal concentration) determined with conventional approaches. The SSF2 medium allowed isolates to grow and form biofilm-like aggregates varying in size and shape between different species. For most isolates cultured in SSF2, a reduced susceptibility to the tested antibiotics was observed when compared to susceptibility data obtained in general media. These data indicate that the *in vitro* SSF2 model could be a valuable addition to evaluate the antimicrobial susceptibility of biofilm-like aggregates in the context of PJI.

**IMPORTANCE:**

Infections after joint replacement are rare but can lead to severe complications as they are difficult to treat due to the ability of pathogens to form surface-attached biofilms on the prosthesis as well as biofilm aggregates in the tissue and synovial fluid. This biofilm phenotype, combined with the microenvironment at the infection site, substantially increases antimicrobial tolerance. Conventional *in vitro* models typically use standard growth media, which do not consider the microenvironment at the site of infection. By replacing these standard growth media with an *in vivo*-like medium, such as the synthetic synovial fluid medium, we hope to expand our knowledge on the aggregation of pathogens in the context of PJI. In addition, we believe that inclusion of *in vivo*-like media in antimicrobial susceptibility testing might be able to more accurately predict the *in vivo* susceptibility, which could ultimately result in a better clinical outcome after antimicrobial treatment.

## INTRODUCTION

Due to an increased life expectancy, the number of total joint replacements, along with the risk on serious complications such as (peri)prosthetic joint infections (PJIs), continues to increase (1, 2). These infections are typically caused by unintentional inoculation during surgery or shortly thereafter and by hematogenous spread from adjacent tissues (3). The infecting pathogens most commonly found in PJI are *Staphylococcus aureus* and coagulase-negative staphylococci (e.g., *Staphylococcus epidermidis*). In addition, a smaller percentage of other microorganisms are also encountered in PJI, including *Streptococcus* spp., *Enterococcus* spp., anaerobes (e.g., *Cutibacterium acnes*), Gram-negative rods such as *Pseudomonas aeruginosa* and *Escherichia coli*, and *Candida* spp. (4, 5). However, a considerable proportion of the cases remain culture negative, even when there are clear signs of infection (6).

PJIs are difficult to treat, as antibiotics often fail to reach the site of infection (7) and because of the ability of microorganisms to form surface-attached biofilms on prostheses (8), as well as biofilm-like aggregates embedded in synovial fluid and tissues (9). These biofilms are very hard to fully eradicate; hence, extraction of the prosthesis is often required (8). Components such as fibrinogen, hyaluronic acid, and albumin, present in human synovial fluid, induce biofilm-like aggregate formation in the synovial cavity (10, 11), and this biofilm phenotype, as well as the specific microenvironment (12), contributes to increased antimicrobial tolerance (13, 14). Several studies reported that biofilm-like aggregates formed by PJI pathogens in synovial fluid show a strongly reduced susceptibility toward antibiotics, both in *in vitro* and in *ex vivo* models (9, 11, 15, 16).

In clinical practice, the antimicrobial susceptibility of a pathogen is typically assessed by determining the minimal inhibitory concentration (MIC) using microbroth dilution or gradient strip methods or by disk diffusion; these assays are typically performed in standard growth media (17, 18). Whereas these tests can accurately predict the susceptibility of planktonic cells, they often fail to predict treatment success as they do not consider the impact of the biofilm phenotype and the infectious microenvironment on the antimicrobial susceptibility (12, 19). Indeed, the growth medium used is of great importance as it impacts gene expression profiles, and gene expression profiles of *S. aureus* differ markedly after cultivation in conventional growth media or in human synovial fluid (20). Furthermore, *S. aureus* gene expression *in vitro* differs from what is observed *in vivo*; e.g., genes involved in siderophore synthesis and multiple known virulence genes are upregulated *in vivo* (21).

One way to mimic the *in vivo* microenvironment *in vitro* is to use growth media that closely resemble the *in vivo* nutritional environment. In the present study, we developed a synthetic synovial fluid (SSF2) medium that simulates the microenvironment within the joints of PJI patients and can easily be integrated into *in vitro* testing. After initial optimization with an *S. aureus* PJI isolate, the model was further validated using multiple PJI isolates. Additionally, MICs and minimal bactericidal concentrations (MBCs) were determined using conventional approaches and compared to biofilm preventing concentrations (BPCs, i.e., the lowest concentration of an antibiotic required to fully prevent formation of a biofilm, including biofilm aggregates, starting from planktonic cells) and minimum biofilm inhibitory concentrations (MBICs, i.e., the lowest concentration of an antibiotic required to fully prevent the further development of a biofilm) (19) obtained in SSF2.

## MATERIALS AND METHODS

### Bacterial isolates, culture conditions, and antibiotics

We included 18 clinical PJI isolates in the present study (Table S1), among which 7 isolates were recovered from PJI patients receiving care at the Ghent University Hospital. *C. acnes* isolates were cultured on reinforced clostridial medium (Lab M, Moss Hall, UK) for 48 h at 37°C under anaerobic conditions. *Candida* spp. were cultured on Sabouraud dextrose medium (Lab M) for 24 h at 37°C under aerobic conditions. The remaining isolates were grown on tryptic soy agar (TSA; Neogen, Heywood, UK), after cultivation at 37°C for 24 h or in Mueller-Hinton Broth (MHB, Lab M) for 16 h at 37°C. For antimicrobial susceptibility testing, the following antibiotics were used: rifampicin, oxacillin, cefazolin, amoxicillin, ceftriaxone, fluconazole, ciprofloxacin (all from Merck Life Science, Darmstadt, Germany), doxycycline (TCI, Europe, Zwijndrecht, Belgium), and vancomycin (Thermo Fisher Scientific, Waltham, MA, USA). For all antibiotics, stock solutions of 5 mg/mL were prepared, except for ciprofloxacin and rifampicin. For ciprofloxacin, a stock solution of 3.2 mg/mL with 70-µL 1-M HCl was prepared, and for rifampicin a stock solution of 0.5 mg/mL with 1% dimethylsulfoxide was prepared. All stock solutions were prepared in MilliQ water, filter sterilized (polyethersulfone (PES), 0.22 µm; VWR, Haasrode, Belgium) and stored at 4°C–7°C for a maximum of 1 week prior to use.

### Development of the SSF1 and SSF2 medium

The composition of a first version of the SSF medium (SSF1) was based on the composition of human synovial fluid obtained from healthy individuals (22–26). Fibrinogen was subsequently added to the medium as it contributes to the synovial fluid-induced aggregate formation (10, 11, 27), leading to SSF2. Optimization was done by assessing the growth of *S. aureus* A1 in both media after 4, 24, 48, and 72 h of incubation at 37°C under aerobic, microaerophilic (3% O_2_, 5% CO_2_, 92% N_2_; Bactrox Hypoxic Chamber; SHEL-LAB, Cornelius, USA) and anaerobic conditions (5% H_2_, 5% CO_2_, 90% N_2_; Bactronez-2 Anaerobic Chamber, SHEL-LAB). Based on these results, the optimal composition (Table 1) and culture conditions for the SSF2 model were selected. A detailed protocol for the preparation of the SSF2 medium can be found in the supplemental data. When *S. aureus* A1 was cultured in SSF2, trypsin was required to disrupt the aggregates. To ensure that trypsin did not affect cell viability, planktonic cultures of all 18 PJI isolates were grown in MHB for 24 h at 37°C and were subjected to either a trypsin treatment (0.25%) or a conventional biofilm disruption by sonicating (5 min) and shaking (5 min, 900 rpm). Subsequently, a dilution series of both replicates (trypsin treatment and conventional biofilm disruption) were plated on the above-mentioned agar media. Colonies were counted after 24 h (48 h for *C. acnes*), and the number of CFU was calculated. Each experiment was conducted in duplicate and repeated three times, each time starting from a fresh pure culture.

**TABLE 1:** Composition of the SSF2 medium

Compound	Concentration
Glucose (g/L)	1
MgSO_4_ (mM)	1
CaCl_2_ (mM)	0.3
Nicotinic acid (mg/L)	2
Thiamine (mg/L)	2
Calcium panthothenate (mg/L)	2
Biotin (mg/L)	0.1
Citric acid (mg/L)	24
Bovine serum albumin (g/L)	25
Hyaluronic acid (1.20–1.80 MDa) (g/L)	3
Fibrinogen (g/L)	0.100
Amino acids (mg/L)	
Alanine	50.0
Arginine	8.9
Asparagine	12.0
Aspartic acid	8.8
Cysteine	20.0
Glutamic acid	38.0
Glutamine	76.0
Glycine	29.0
Histidine	18.0
Isoleucine	13.0
Leucine	27.0
Lysine	35.0
Methionine	4.3
Phenylalanine	16.0
Proline	28.0
Serine	20.0
Threonine	19.0
Tryptophan	14.0
Tyrosine	14.0
Valine	2.7.0
Trace elements (mg/mL)	
FeCl_3_	4.98
ZnCl_2_	0.89
CuCl_2_	0.13
CoCl_2_	0.09996
H_3_BO_3_	0.0992
MnCl_2_	0.016
M9 salts (g/L)	
Na_2_HPO_4_·2H_2_O	7.52
KH_2_PO_4_	3.0
NaCl	0.5
NH_4_Cl	0.5

### Evaluation of aggregate formation using light microscopy

Isolates were inoculated in 1 mL of SSF2 in a 24-well plate (F-bottom; Greiner Bio-One, Frickenhausen, Germany) with a final inoculum of 5 × 10^7^ CFU/mL. After 24 h and 48 h of incubation at 37°C under microaerophilic (3% O_2_) or anaerobic conditions, aggregate formation was evaluated using the EVOS FL Auto Imaging System (Life Technologies, Carlsbad, CA, USA) equipped with a 20 x objective.

### Evaluation of aggregate formation in SSF2 using confocal laser scanning microscopy

High-resolution imaging by confocal laser scanning microscopy (CLSM) was carried out on aggregates formed in SSF2 by a selection of staphylococci (*S. aureus* SAU060112, *S. aureus* A1, *Staphylococcus capitis* subsp. *capitis* CCUG 39451, *S. epidermidis* HD05-1 ST2). The selected strains were grown for 24 h in Luria-Bertani (LB) medium (VWR international, Radnor, Pennsylvania, USA) at 37°C under shaking conditions (200 rpm). The cultures were subsequently inoculated in 200 µL of SSF2 in a µ-plate 96 Well Round Glass Bottom (Ibidi GmbH, Grädelfing, Germany) with a final inoculum of 2.6 × 10^8^ CFU/mL. After 24 h of incubation at 37°C under static, aerobic conditions, the biofilms were stained by adding Live/Dead staining (Thermo Fisher Scientific) to a total concentration of 0.3 µL/mL of SYTO9 and propidium iodide (PI). After incubation for 15 min in the dark, the stained biofilms were imaged using a Zeiss LSM880 confocal microscope with 63 x oil-based objective (Plan-Apochromat 63 x/1.4 Oil DIC M27, Carl Zeiss) and an Airyscan Detector (Carl Zeiss NV, Zaventem, Belgium). Images were acquired with a resolution of 85 nm in the x- and y-direction and 182 nm in the z-direction in the FastAiryscan mode using 488 nm and 561 nm lasers for the SYTO0 and PI respectively.

### Biofilm quantification in SSF2 model

Isolates were grown in SSF2 in a 96-well plate (U-bottom, Greiner Bio-One) with a final inoculum of 5 × 10^7^ CFU/mL in a volume of 100 µL and incubated at 37°C under microaerophilic (3% oxygen) or anaerobic conditions for 24 or 48 h. Subsequently, aggregates were disrupted using a 0.25% trypsin EDTA solution (Life Technologies). To this end, the supernatant was removed after centrifuging the plates for 15 min at 3,700 rpm; 100 µL of trypsin was added to the pelleted aggregates; and the plates were incubated for 20 min at 37°C. Thereafter, the plates were centrifuged again for 15 min at 3,700 rpm. Trypsin was then removed and the bacterial cells were resuspended in physiological saline and dilution series were plated on the above-mentioned agar media. Colonies were counted after 24 h of incubation (48 h for *C. acnes*), and the number of CFU was calculated. Each experiment was conducted in duplicate and repeated three times, each time starting from a fresh pure culture.

### Determination of MICs and MBCs

The MIC and MBC of all isolates were determined according to European Committee on Antimicrobial Susceptibility Testing guidelines (17) for a broad range of relevant antibiotics commonly used in the treatment of PJI (Table S2) (28). For the MIC determination, an inoculum of 5 × 10^5^ CFU/mL was incubated for 24 h at 37°C in the presence of a serial dilution of antibiotics in a final volume of 200 µL under static conditions. MBCs were determined by plating the entire contents of the wells containing the MIC and the four subsequent wells containing higher antibiotic concentrations on the above-mentioned agar media. After 24 h of incubation at 37°C, the lowest antibiotic concentration that prevented growth was considered as MBC. The highest antimicrobial concentrations tested were based on solubility and clinical relevance and are shown in Table S3. Each experiment was conducted in duplicate and repeated three times, each time starting from a fresh pure culture. Median values of these triplicates were used as MIC and MBC values for subsequent data analysis.

### Determination of BPCs and MBICs

For the BPC determination, overnight cultures were diluted to a final inoculum of 5 × 10^5^ CFU/mL in a 96-well plate (U-bottom) containing serial twofold dilutions of the different antibiotics in SSF2, with a final volume of 200 µL. Non-treated controls (containing only bacteria in SSF2) and blanks (containing only SSF2) were included in each experiment. After 24 h of incubation at 37°C, under microaerophilic (3% oxygen) conditions, biofilms were disrupted using trypsin treatment as described above. For the determination of the MBIC, biofilms were first grown in SSF2 for 24 h at 37°C, under microaerophilic (3% oxygen) conditions in the absence of antibiotics. The biofilms were then treated with serial twofold dilutions of the different antibiotics (prepared in SSF2 in a separate 96-well plate) and subsequently incubated for another 24 h. Staining with resazurin (CellTiter-Blue [CTB]) (Promega, Leiden, The Netherlands) was used to determine the BPC and MBIC. The resazurin solution was prepared by diluting 2.1-mL stock solution of CTB with 10.5-mL physiological saline (0.9% NaCl). One hundred twenty microliters of this CTB solution was added to each well. Plates were covered from the light and incubated for 20 min–1 h, depending on species, at 37°C under static conditions. Using a Viktor Nivo plate reader (PerkinElmer, Waltham, USA), the fluorescence was measured (excitation wavelength: 560 nm and emission wavelength: 590 nm). The BPC and MBIC were defined as the lowest concentration of an antibiotic that reduces the resazurin-derived fluorescence with at least 90% compared to a non-treated control after 24 h of exposure to the antibiotics. To ensure that the resazurin staining is a reliable alternative to determine these biofilm parameters, a correlation analysis was performed on the fluorometric values and CFU counts of six isolates that were treated with oxacillin (ie., *S. aureus* A1, SAU060112, IDRL-9783, IDRL-10982, *Staphylococcus lugdunensis* CCUG52060, and *S. capitis* subsp. *capitis* CCUG39451). More specifically, after measuring the resazurin-derived fluorescence of all conditions, the content of all wells was plated in parallel on TSA. After 24 h of incubation at 37°C, colonies were counted and the number of CFU was calculated. All experiments were performed in duplicate and repeated three times, each time starting from a fresh pure culture.

### Statistical analysis

To check whether CFU counts obtained after incubation in the two different SSF media differed from each other and from the CFU counts obtained after trypsin treatment, a one-way analysis of variance (ANOVA) test (with Bonferroni correction) was performed for normally distributed data, and the non-parametric one-way ANOVA Kruskal-Wallis test was used for non-normally distributed data. To check whether CFU counts were affected by trypsin treatment, an independent sample *t*-test was performed for normally distributed data, and the non-parametric Mann-Whitney *U* test was used for non-normally distributed data. Normal distribution was assessed using the Shapiro-Wilk test. To determine whether CTB-derived fluorescence can be used as an alternative to plating for determining the number of CFU, Kendall’s tau correlations (*r*_*τ*_) between fluorescence and CFU counts were calculated (29, 30). Values for non-treated controls were also included. Datapoints below the limit of detection, i.e., fluorescence values equal to 1 and CFU counts less or equal to 10^2^ CFU/mL, were excluded. All statistical analyses were performed using SPSS Statistics software (version 28, IBM, New York, USA).

## RESULTS

### Fibrinogen as a key component in synovial fluid-induced aggregate formation of *S. aureus*

**Fig 1:**
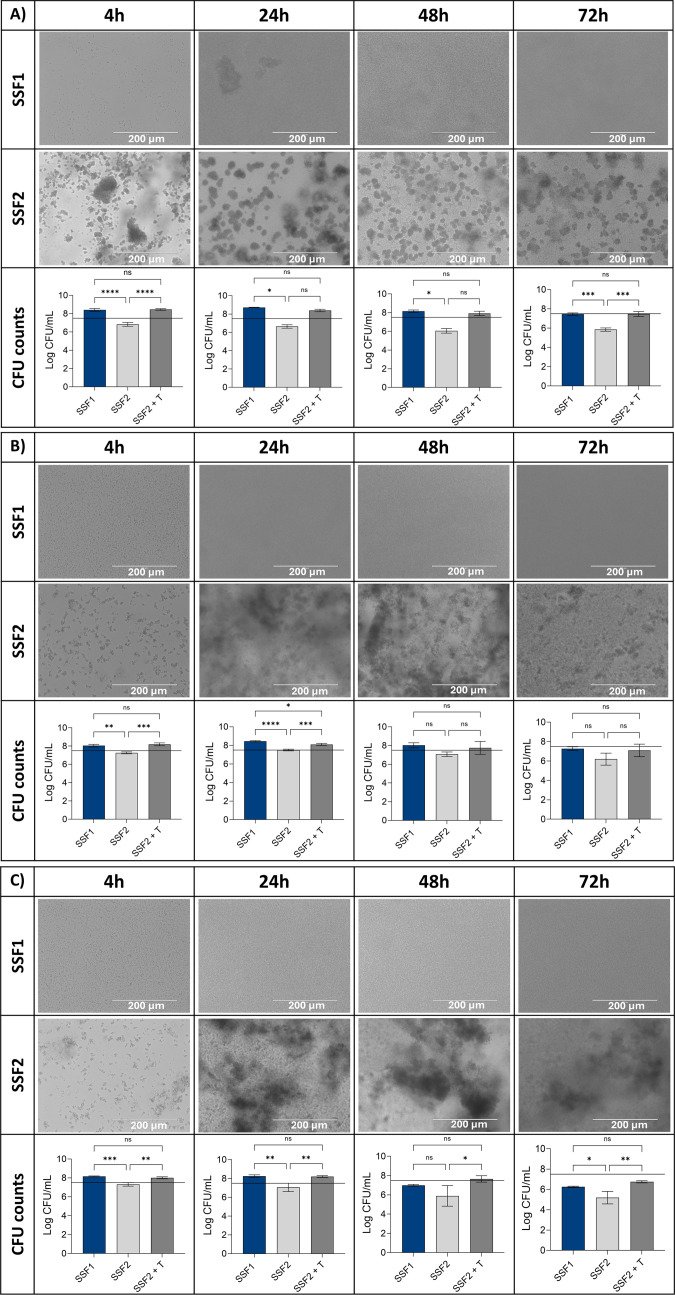
Light microscopy images of clinical *S. aureus* isolate A1 cultured in SSF without Fg (SSF1) and SSF with Fg (SSF2) under aerobic (**A**), microaerophilic (3% O_2_) (**B**), and anaerobic (**C**) conditions, evaluated at four different timepoints. The bars represent the average number (*n* = 3) of CFUs recovered after conventional biofilm disruption for biofilms grown in SSF1 (blue) and SSF2 (light gray) or after biofilm disruption using trypsin (0.25%) after growth in SSF2 (dark gray). Error bars indicate standard deviation; black line indicates the inoculum of 5 × 10^7^ CFU/mL. *P < 0.05, **P < 0.01, ***P < 0.001. ns, not significant (*P* ≥ 0.05).

To assess the effect of including fibrinogen in the medium, growth and aggregate formation of *S. aureus* A1 were checked after 4, 24, 48, and 72 h of incubation in SSF1 (without fibrinogen) and SSF2 (supplemented with fibrinogen), at different oxygen levels (Fig. 1). *S. aureus* A1 showed growth in SSF1 at all oxygen levels after 4 and 24 h of incubation. Under microaerophilic and anaerobic conditions, CFU counts dropped below the inoculum size after 48 h of incubation in SSF1. After 72 h of incubation in SSF1, CFU counts dropped below the inoculum size at all oxygen levels tested. Light microscopy images show growth at all oxygen levels, at all timepoints; however, no aggregate formation was observed in SSF1. Surprisingly, when CFU counts were determined after incubation in SSF2 at varying oxygen levels, no growth was observed; however, light microscopy indicated the presence of *S. aureus* A1 aggregates in SSF2. We hypothesized that the intracellular interactions within the aggregates were too strong to disrupt the aggregates using conventional biofilm disruption approaches, and for this reason, a trypsin treatment was introduced. Following trypsin treatment, CFU counts after growth in SSF2 no longer significantly differed from the CFU counts found after incubation in SSF1. As a consequence, trypsin treatment was introduced in all further experiments to quantify aggregate formation in SSF2. We checked whether trypsin treatment affected the cell viability of all PJI isolates included in this study, but no significant differences were observed between CFU counts obtained after conventional biofilm disruption when compared to CFU counts obtained after trypsin treatment (Fig. S1).

### SSF2 model allows growth and aggregate formation of different PJI isolates

**Fig 2:**
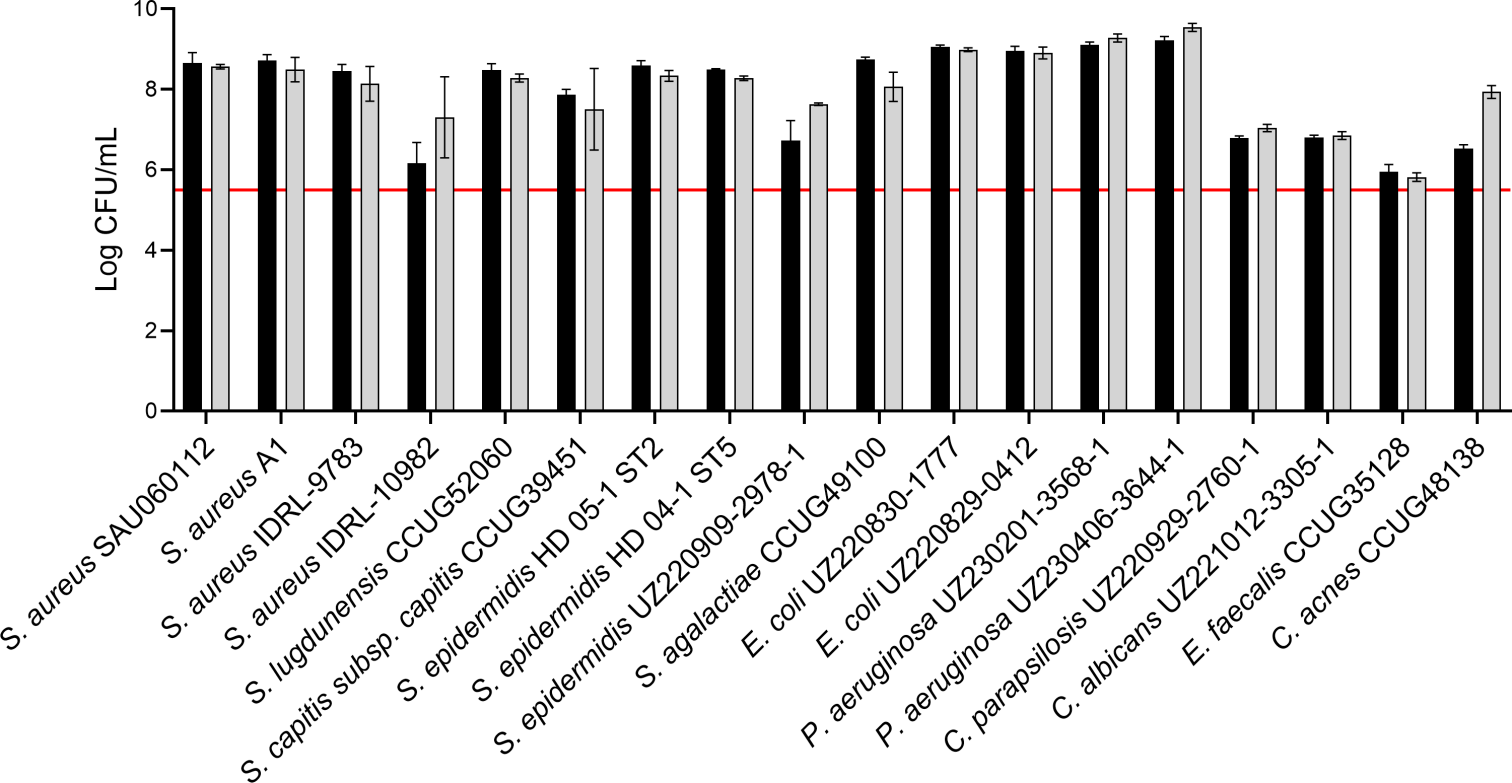
Log CFU per milliliter values of 18 clinical PJI isolates after 24 h (black bars) and 48 h (gray bars) of incubation in SSF2 under microaerophilic conditions (3% O_2_), except for *C. acnes* (anaerobic conditions). Inoculum size: 5 × 10^5^ CFU/mL (red line). Data shown are mean values of biological replicates; error bars represent standard deviations (*n* = 3).

**Fig 3:**
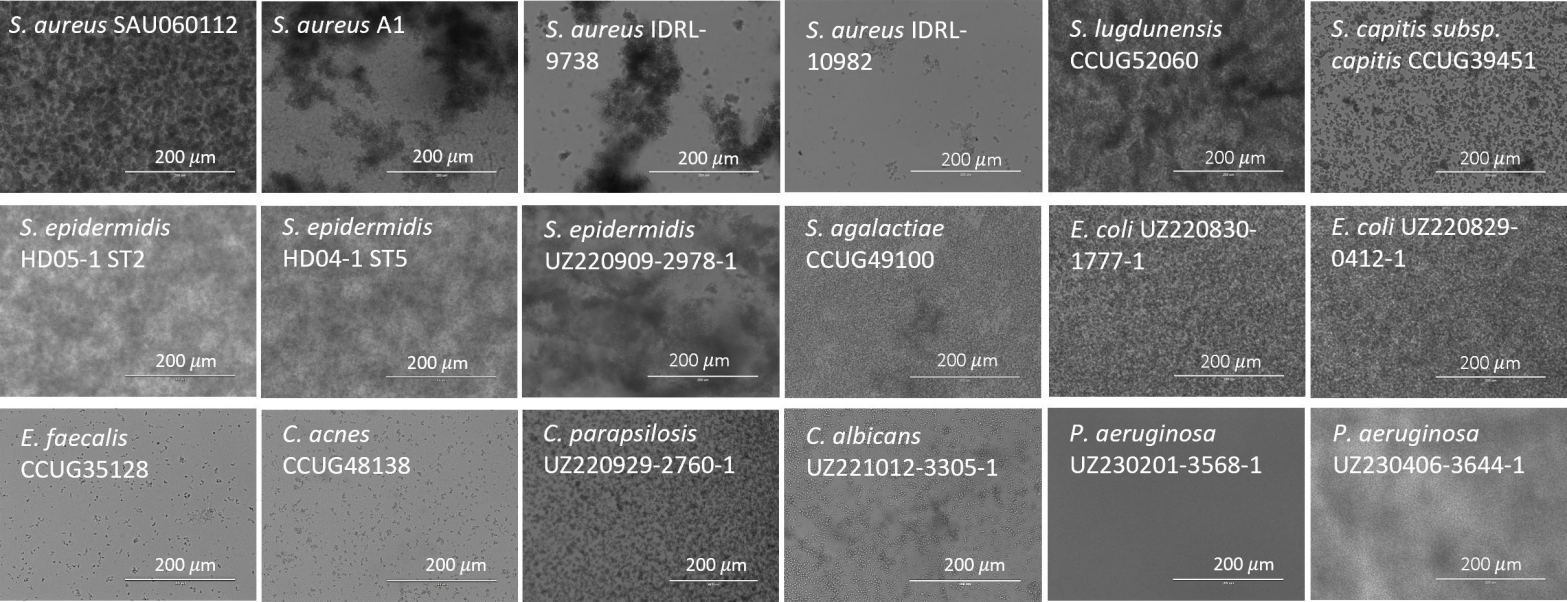
Light microscopy images of 18 clinical PJI isolates after 24 h of incubation in SSF2 under microaerophilic conditions (3% O_2_), except for *C. acnes* (anaerobic conditions). Scale bars 200 µm.

**Fig 4:**
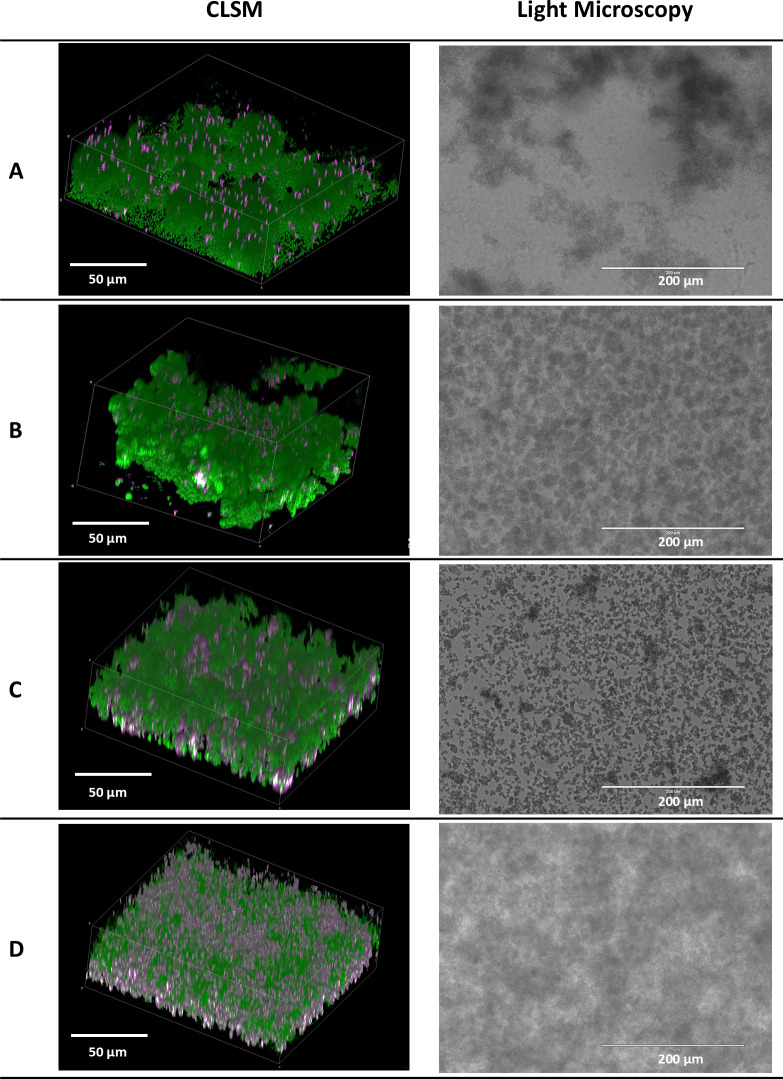
CLSM (left) and light microscopy (right) images of four of the staphylococci. (**A**) *S. aureus* A1, (**B**) *S. aureus* SAU060112, (**C**) *S. capitis* subsp. *capitis* CCUG 39451, and (**D**) *S. epidermidis* HD05-1 ST2, after 24 h of incubation in SSF2. CLSM: scale bars 50 µm; light microscopy: scale bars 200 µm. The contrast was adjusted to improve visualization. The light microscopy images shown (on the right) are the same as the ones shown in Fig. 3 for the four strains mentioned.

Subsequently, growth and aggregate formation of the 18 isolates included in this study were assessed in the SSF2 medium. Fifteen out of the 18 isolates grew well in SSF2 after 48 h of incubation under microaerophilic conditions (anaerobic conditions for *C. acnes*), with an increase of at least 2 log CFU/mL (Fig. 2) compared to the inoculum. The Gram-negative bacteria included, as well as some of the staphylococci (*S. aureus* SAU060112 and A1, *S. epidermidis* HD05-1 ST2 and HD04-1 ST5), showed the strongest increase of at least 3 log CFU/mL after 48 h of incubation compared to the inoculum. For the other staphylococci, *Streptococcus agalactiae* and *C. acnes*, an increase between 2.0 and 2.5 log CFU/mL was observed after 48 h of incubation. The growth of the *Candida* spp. was less pronounced (increase of 1.5 CFU/mL). No growth was observed only for *Enterococcus faecalis* after 48 h. Light microscopy showed that all staphylococci form large, clustered aggregates, except *S. aureus* IDRL-10982, for which a lower number of small aggregates was observed (Fig. 3). The number of aggregates formed by *E. faecalis* and *C. acnes* was lower, and their volume was less dense than for the other isolates. For the remaining isolates, a high density of cells was observed, and aggregates differed in size and shape from the staphylococcal aggregates. *P. aeruginosa* UZ230201-3568-1 was the only isolate for which no aggregate formation was observed (Fig. 3). A more detailed morphological evaluation was carried out on a selection of staphylococci cultured in SSF2, using CLSM following Live/Dead staining (Fig. 4). Overall, the CLSM images corroborate the findings using light microscopy. *S. aureus* A1, *S. aureus* SAU060112, and *S. capitis* formed dense clusters interspersed with regions of lower cell density, whereas *S. epidermidis* formed a confluent layer covering the entire surface. The biofilms consisted mostly of live cells, with dead cells primarily found in the bottom layer of the *S. capitis* and *S. epidermidis* biofilms.

### Resazurin staining as a reliable alternative quantification approach

**Fig 5:**
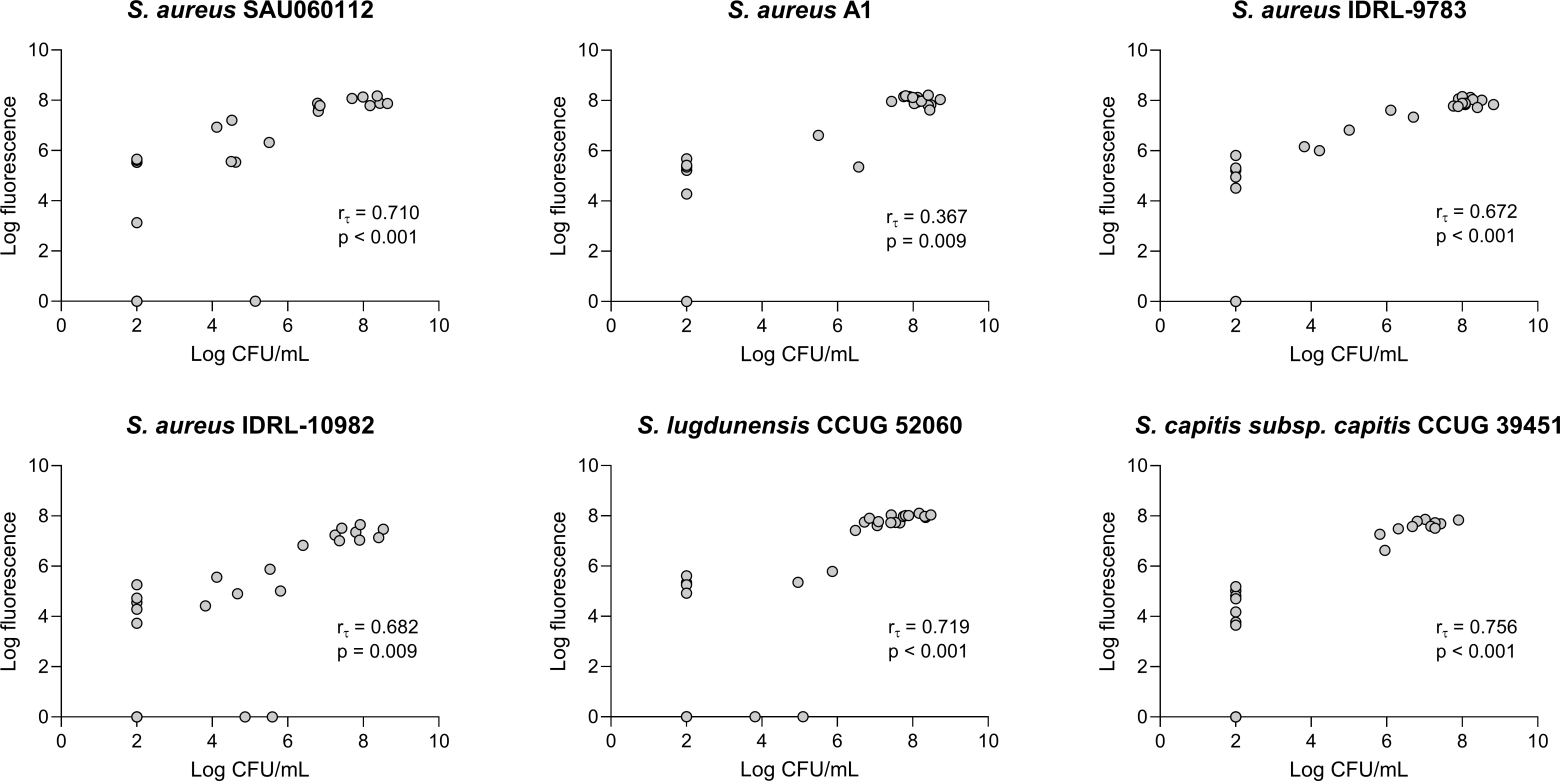
Relation between log CFU per milliliter values and log fluorescence values after resazurin staining. Data are shown for all the replicates tested (*n* = 3).

Subsequently, we investigated whether resazurin could be used for quantification of growth in SSF2. This was done using biofilm aggregates formed by *S. aureus* A1, SAU060112, IDRL-9783, IDRL-10982, *S. lugdunensis* CCUG52060, and *S. capitis* subsp. *capitis* CCUG39451 that were exposed to increasing oxacillin concentrations (ranging from 0.25 to 256 µg/mL). A significant (*P* < 0.05) and strong (*r*_*τ*_ ≥0.3) correlation between the fluorescence values and CFU values (both log-transformed) was found (Fig. 5) (30).

### Biofilm susceptibility parameters (BPC and MBIC) differ markedly from conventional susceptibility parameters (MIC and MBC)

**Fig 6:**
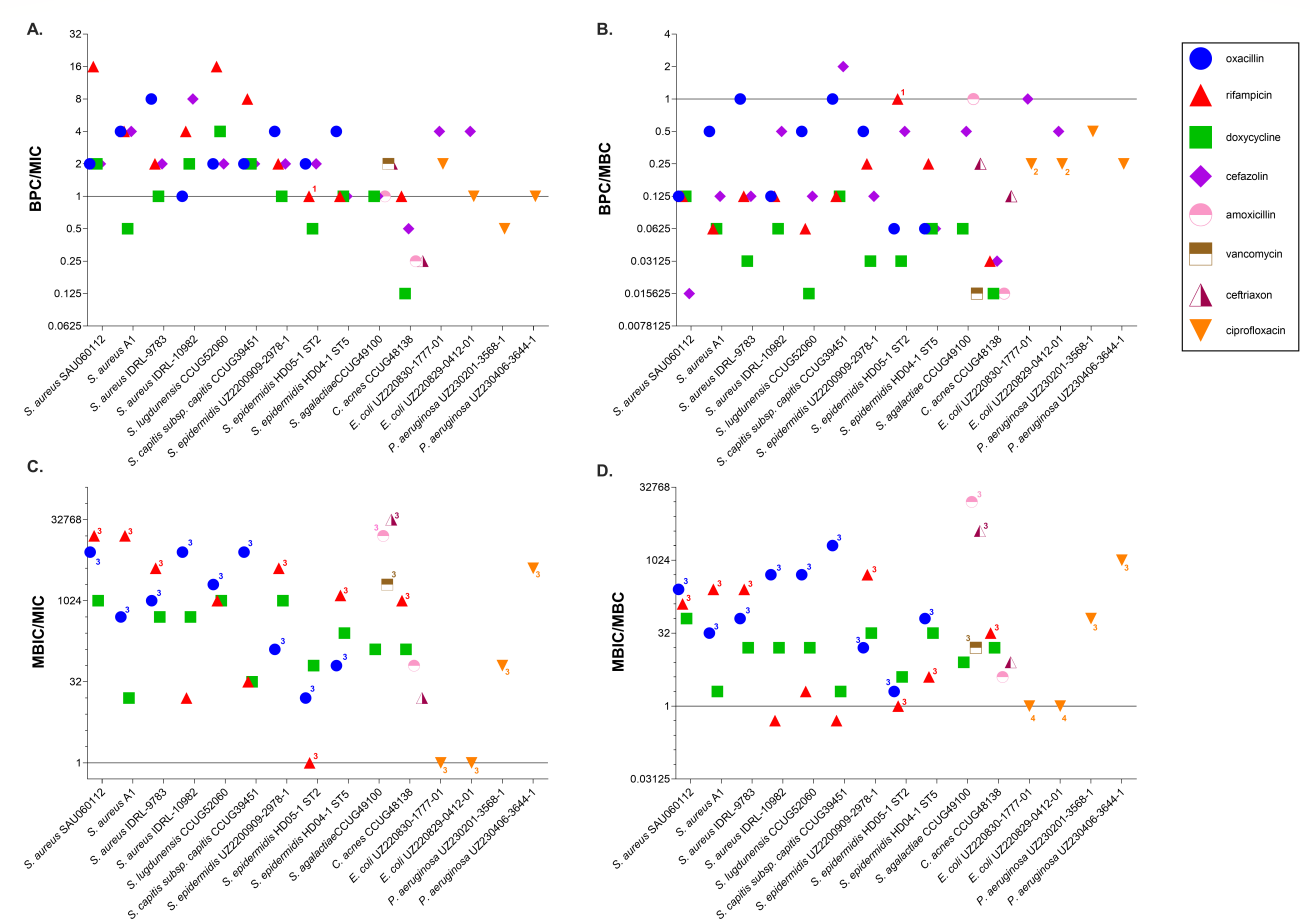
BPC:MIC ratios (**A**), BPC:MBC ratios (**B**), MBIC:MIC ratios (**C**), and MBIC:MBC ratios (**D**) for all antibiotics tested. The solid black line indicates a ratio of 1. Data points for which an exact ratio could not be determined because one or both parameters are outside the testing range are labeled: (1) BPC >testing range, (2) MBC >testing range, (3) MBIC >testing range, and (4) MBIC >testing range and MBC <testing range.

MIC, MBC, BPC, and MBIC values of the different antibiotics for the 18 PJI isolates investigated are shown in Table 2. For cefazolin, the MBIC was not determined, as this antibiotic is traditionally administered as prophylactic before surgery; hence, only the BPC was determined. Due to intrinsic resistance of *P. aeruginosa* and *Candida* spp. to cefazolin, corresponding BPCs and MBICs were not determined. Likewise, given that the MIC and MBC of fluconazole for both *Candida* spp. strains exceeded the highest test concentration (512 µg/mL), the BPC and MBIC were not determined. Finally, for *E. faecalis*, BPCs and MBICs were not determined as this isolate did not grow in the SSF2 medium. The biofilm susceptibility data are expressed relative to the planktonic susceptibility data (i.e., relative to MIC and MBC) (Fig. 6). In some cases, an exact ratio could not be determined as at least one of the parameters had a value that was outside the testing range (i.e., higher than the highest test concentration or lower than the lowest test concentration).

**TABLE 2:** Overview of median MICs, MBCs, BPCs, and MBICs (in µg/mL) of the different antibiotics for the 18 clinical PJI isolates investigated^*a*^

	MIC	MBC	BPC	MBIC
Oxacillin				
*S. aureus* SAU060122	0.25	4	0.5	>1,024
*S. lugdunensis* CCUG 52060	1	2	1	>1,024
*S. capitis* subsp. *capitis* CCUG 39451	0.25	0.5	0.5	>1,024
*S. aureus* A1	4	32	16	>1,024
*S. aureus* IDRL-9783	2	16	16	>1,024
*S. aureus* IDRL-10982	0.25	2	0.25	>1,024
*S. epidermidis* UZ220909-2978-1	8	64	32	>1,024
*S. epidermidis* HD05-1 ST2	64	512	32	>1,024
*S. epidermidis* HD04-1 ST5	16	256	64	>1,024
Rifampicin				
*S. aureus* SAU060122	0.001953	0.25	0.03125	>32
*S. lugdunensis* CCUG 52060	0.000977	0.25	0.015625	0.5
*S. capitis* subsp. *capitis* CCUG 39451	0.003906	0.25	0.03125	0.125
*S. aureus* A1	0.001953	0.125	0.0078125	>32
*S. aureus* IDRL-9783	0.0078125	0.125	0.015625	>32
*S. aureus* IDRL-10982	0.003906	0.125	0.015625	0.0625
*S. epidermidis* UZ220909-2978-1	0.0078125	0.0625	0.015625	>32
*S. epidermidis* HD05-1 ST2	32	32	>32	>32
*S. epidermidis* HD04-1 ST5	0.025	0.5	0.03125	>32
*C. acnes* CCUG 48138	0.03125	1	0.015625	>32
Doxycyclin				
*S. aureus* SAU060122	0.25	4	0.5	256
*S. lugdunensis* CCUG 52060	0.125	8	0.125	128
*S. capitis* subsp. *capitis* CCUG 39451	4	64	8	128
*S. aureus* A1	16	128	8	256
*S. aureus* IDRL-9783	0.25	8	0.25	128
*S. aureus* IDRL-10982	0.25	8	0.5	128
*S. epidermidis* UZ220909-2978-1	0.125	4	0.125	32
*S. epidermidis* HD05-1 ST2	2	32	1	128
*S. epidermidis* HD04-1 ST5	0.25	4	0.25	128
*S. agalactiae* CCUG 49100	8	128	8	1,024
*E. faecalis* CCUG 35182	4	128	ND	ND
*C. acnes* CCUG 48138	2	16	0.25	256
Cefazolin				
*S. aureus* SAU060122	0.25	32	0.5	ND
*S. lugdunensis* CCUG 52060	0.5	8	1	ND
*S. capitis* subsp. *capitis* CCUG 39451	1	1	2	ND
*S. aureus* A1	1	32	4	ND
*S. aureus* IDRL-9783	2	32	4	ND
*S. aureus* IDRL-10982	0.5	8	4	ND
*S. epidermidis* UZ220909-2978-1	4	64	8	ND
*S. epidermidis* HD05-1 ST2	64	256	128	ND
*S. epidermidis* HD04-1 ST5	4	64	4	ND
*S. agalactiae* CCUG 49100	0.25	0.5	0.25	ND
*E. faecalis* CCUG 35182	32	1024	ND	ND
*C. acnes* CCUG 48138	0.5	8	0.25	ND
*E. coli* UZ300822-0412-1	8	32	32	ND
*E. coli* UZ290822-1777-1	8	64	32	ND
*P. aeruginosa* UZ230201-3568-1	>1,024	>1,024	ND	ND
*P. aeruginosa* UZ230406-3644-1	>1,024	>1,024	ND	ND
*Candida parapsilosis* UZ220929-2760-1	>1,024	>1,024	ND	ND
*Candida albicans* UZ221012-3305-1	>1,024	>1,024	ND	ND
Amoxicillin				
*S. agalactiae* CCUG 49100	0.0625	0.0625	0.0625	>1,024
*E. faecalis* CCUG 35182	4	4	ND	ND
*C. acnes* CCUG 48138	0.25	4	0.0625	16
Vancomycin				
*S. agalactiae* CCUG 49100	0.5	64	1	>1,024
*E. faecalis* CCUG 35182	1	1	ND	ND
Ceftriaxon				
*S. agalactiae* CCUG 49100	0.03125	0.25	0.0625	>1,024
*E. faecalis* CCUG 35182	512	>1,024	ND	ND
*C. acnes* CCUG 48138	0.5	1	0.125	8
Ciprofloxacin				
*E. coli* UZ300822-0412-1	128	>1,024	256	>1,024
*E. coli* UZ290822-1777-1	256	>1,024	256	>1,024
*P. aeruginosa* UZ230201-3568-1	16	16	8	>1,024
*P. aeruginosa* UZ230406-3644-1	0.25	1	0.25	>1,024
Fluconazole				
*C. parapsilosis* UZ220929-2760-1	>512	>512	ND	ND
*C. albicans* UZ221012-3305-1	>512	>512	ND	ND

^*a*^ ND, not determined.

In most cases, the BPC:MIC ratio was higher than 1 (64%) (fold difference between 2 and 16) (Fig. 6A). In 24% of the susceptibility tests, the BPC:MIC ratio was equal to 1 (i.e., the BPC equaled the MIC). The BPC was lower than the MIC in 12% of the cases, more specifically for *S. aureus* A1 and *S. epidermidis* HD05-1 ST2 and doxycycline (BPC twofold lower than MIC): the BPCs of all antibiotics tested for *C. acnes* CCUG48138 were smaller than corresponding MICs (two- to fourfold difference), and finally, the BPC of ciprofloxacin for *P. aeruginosa* UZ230201-3568-1 was two times lower than its MIC. The BPC was lower than the MBC in 86% of the cases, and the extent of this difference varied between 2- and 64-fold (Fig. 6B). The BPCs of oxacillin for *S. capitis* subsp. *capitis* CCUG39451 and *S. aureus* IDRL-9783, of rifampicin for *S. epidermidis* HD05-1 ST2, of amoxicillin for *S. agalactiae* CCUG49100, and of cefazolin for *E. coli* UZ220830-1777-1 were equal to the MBC (12%). The BPC of cefazolin exceeded the MBC (2%) (twofold difference) only for *S. capitis* subsp. *capitis*.

In the majority of the cases, the MBIC exceeded the MIC (91.5%), except for the combinations rifampicin/*S. epidermidis* HD05-1 ST2, ciprofloxacin/*E. coli* UZ220830-1777-01, and ciprofloxacin/*E. coli* UZ220829-0412-01 (Fig. 6C). For these combinations (8.5%), at least one of the susceptibility parameters was outside the testing range, and as a consequence, an exact ratio could not be determined. The extent to which the MBIC differed from the MIC in the other cases was much larger than the difference between BPC and MIC, ranging from 16- to 16,384-fold differences. The MBIC exceeded the MBC in most of the cases (86.5%), and the extent of this difference varied between 2- and 4,096-fold (Fig. 6D). However, for 13.5% of the combinations (rifampicin/*S. epidermidis* HD05-1 ST2, rifampicin/*S. lugdunensis* CCUG52060, rifampicin/*S. aureus* IDRL-9783 ciprofloxacin/*E. coli* UZ220830-1777-01, and ciprofloxacin/*E. coli* UZ220829-0412-01), at least one of the susceptibility parameters was outside the testing range, and as a consequence, an exact ratio could not be determined.

## DISCUSSION

The aim of the present study was to develop an SSF model that closely mimics the nutritional microenvironment within the joints of patients with PJI and to validate this model in terms of growth, aggregate formation, and antimicrobial susceptibility. To do so, growth and aggregate formation of the clinical isolate *S. aureus* A1 were determined after incubation in SSF media with and without fibrinogen, at different oxygen levels, and with/without trypsin treatment. Aggregate formation of *S. aureus* A1 was only observed in the SSF2 medium supplemented with fibrinogen, indicating fibrinogen is crucial for the aggregatory phenotype of bacteria in synovial fluid. Previous studies have shown that the presence of fibrinogen in human synovial fluid causes bacterial aggregation (15, 31), and proteolytic enzymes are required to disrupt these aggregates (10). Indeed, after treating the aggregates with trypsin, a statistically significant increase in CFU counts was observed. For this reason, we selected the SSF2 medium with fibrinogen and included trypsin treatment in further experiments to disperse aggregates before quantifying bacteria post-incubation. At the site of infection, bacteria are typically exposed to hypoxic conditions, impacting bacterial properties (32). Therefore, microaerophilic conditions (3% O_2_) were chosen to better mimic the *in vivo* situation for further experiments (except for *C. acnes*, for which anaerobic conditions were used). Light microscopy demonstrated that nearly all isolates formed aggregates after incubation in SSF2, varying in size, shape, and density across isolates. Only *P. aeruginosa* UZ230201-3568-1 did not form aggregates, possibly due to its mucoid phenotype. Furthermore, analysis of the three-dimensional structures of a selection of staphylococci by CLSM confirmed the light microscopy findings. Whereas multiple studies showed that synovial fluid induced aggregation of *S. aureus* (31, 33), only a few studies demonstrated aggregate formation in synovial fluid of other PJI pathogens, including coagulase-negative staphylococci, *E. coli*, and *P. aeruginosa* (9, 16). CFU counts indicated all isolates grew well in SSF2, except for *E. faecalis* CCUG35128. Nevertheless, light microscopy revealed small aggregates of *E. faecalis* CCUG35128 were present after 24 h of incubation in SSF2. Recently, a study demonstrated that simulated synovial fluid induces aggregate formation but reduces growth of *E. faecalis* when compared to standard media. These findings suggest synovial fluid media compromise the viability of *E. faecalis* or prohibit acquisition of critical nutrients for their survival (34). Resazurin staining was used to determine the BPC and MBIC, offering a rapid alternative to plating. Our results show there is a significant and strong correlation between CFU counts and resazurin-derived fluorescence for staphylococci, which is consistent with findings from prior research (35, 36). Over the past years, multiple studies on susceptibility testing in synovial fluid media have been reported, all concluding that the use of synovial fluid media alters the antimicrobial efficacy *in vitro* when compared to conventional susceptibility testing (9, 11, 16, 20, 34, 37). Comparing results across studies is difficult because different types of synovial fluid media were used. Besides human and animal synovial fluid (9, 11, 16, 20, 37) that are typically used for *in vitro* experiments, other synthetic synovial fluid media have been developed to better mimic the *in vivo* microenvironment in PJI, including simulated synovial fluid (34) and artificial synovial fluid (38). However, both of these lack fibrinogen, while the latter medium, derived from human plasma, also lacks hyaluronic acid, essential for the viscous properties of synovial fluid. In the present study, we have confirmed that fibrinogen plays an essential role in synovial fluid-induced aggregation and therefore should be included in synthetic media mimicking synovial fluid. One of the limitations of this medium is the fact that its composition does not perfectly reflect the composition of synovial fluid encountered in the diseased joints of PJI patients. It is well established that the proteome (including albumin and fibrinogen expression) (39, 40) and hyaluronic acid structure (41) in healthy joints is distinct from the ones observed in osteoarthritic joints and fluctuates in osteoarthritic joints as well (39). This makes that these concentrations are hard to duplicate *in vitro*. Therefore, we opted for a composition based on healthy synovial fluid, incorporating only the minimal fibrinogen concentration required for bacterial aggregation, as this aligns with our research focus. As mentioned above, it is also possible to use human or animal synovial fluid, which may more closely resemble the *in vivo* proteome. Nevertheless, their use comes with several disadvantages, such as the risk of contamination and the burden of ethical concerns. In addition, the exact composition of these synovial fluids is not known and is likely to vary from individual to individual, making standardization very difficult (42, 43). Using the resazurin-based viability staining and SSF2, we observed BPC values were mostly higher than MIC values, while this was always the case for MBIC values. BPC values were almost always lower than the MBC, while MBIC values were consistently higher than the MBC (with a few exceptions). Notably, for *C. acnes* CCUG48138, the BPC of the five antibiotics tested was consistently lower than the MIC; however, the corresponding MBIC values were much higher than MIC (and BPC) values. To date, only data on surface-attached *C. acnes* biofilms, established in general growth media in *in vitro* models, are available. These data indicate that *C. acnes* biofilms contribute to a reduced antimicrobial susceptibility *in vivo* (44). Yet, why antimicrobial concentrations to prevent biofilm formation by *C. acnes* in our SSF2 medium are lower than the one to inhibit planktonic growth remains unclear. For the staphylococci included, the BPC:MIC ratios and MBIC:MIC ratios of rifampicin were not significantly lower than the ratios of the other antibiotics tested, while rifampicin is considered as a “biofilm-active” antibiotic, typically recommended for the treatment of difficult-to-treat PJI caused by staphylococci (45). Nevertheless, there has been some debate concerning the added value of rifampicin for the treatment of PJI (46, 47). It should be noted that exact MBIC values could not be determined in several cases, as they exceeded the highest test concentrations, which were already much higher than concentrations achievable *in vivo*.

In summary, by mimicking essential features of the nutritious microenvironment we can replicate the aggregatory phenotype of multiple pathogens in the context of PJI. Moreover, it is anticipated that the response of these PJI pathogens to antimicrobials using this approach is more predictive of real-life susceptibility. Whether the use of the SSF2 medium as an alternative culture medium for *in vitro* susceptibility testing will facilitate antimicrobial treatment selection and ultimately lead to a better clinical outcome remains to be evaluated by follow-up studies, including a larger number of isolates and linking this to clinical data. Studies investigating the relationship between biofilm susceptibility and clinical outcomes in staphylococcal PJI have yielded varied results. Increased antimicrobial tolerance observed *in vitro* does not consistently correlate with poorer clinical outcomes and vice versa (48–50). However, it is important to note that these studies were performed using the Calgary Biofilm device, where biofilms are grown on the surface of plastic pegs in standard growth media (51), which does not reflect the situation *in vivo*. The *in vivo*-like SSF2 medium developed in the present study enables the investigation of the biofilm-like aggregation of a variety of PJI pathogens, and it offers a robust alternative to conventional *in vitro* models for testing the antimicrobial susceptibility of these aggregates.

## SUPPLEMENTAL MATERIAL

Supplemental materials spectrum.01980-24-s0001.pdf Supplemental tables, figure, and full protocol.

ASM does not own the copyrights to Supplemental Material that may be linked to, or accessed through, an article. The authors have granted ASM a non-exclusive, world-wide license to publish the Supplemental Material files. Please contact the corresponding author directly for reuse.
