# Supplementary material for: A novel synthetic synovial fluid model for investigating biofilm formation and antibiotic susceptibility in prosthetic joint infections
Source: Microbiol Spectr. 2024 Nov 29;13(1):e01980-24. doi: 10.1128/spectrum.01980-24 (PMC11705890; doi:10.1128/spectrum.01980-24)
Supplement: Supplemental materials — Supplemental tables, figure, and full protocol. [file spectrum.01980-24-s0001.pdf]

Supplementary data

Table S1. Isolates included in the present study.

Species	Designation	Origin	Source (Reference)
S. aureus	SAU060112	Prosthetic components, psoriatic arthritis	T.R. Thomsen (Xu et al. [1])
	A1	PJI knee	Own isolate
	IDRL-9783	PJI knee	R. Patel (Masters et al. [2])
	IDRL-10982	PJI hip	R. Patel (Masters et al. [2])
S. lugdunensis	CCUG52060	Human synovial fluid	CCUG
S. capitis subsp. capitis	CCUG39451	Human joint, arthritis	CCUG
S. epidermidis	HD05-1 ST2	Human synovial fluid, knee	H. Rohde (Both et al. [3])
	HD04-1 ST5	Human synovial fluid, hip	H. Rohde (Both et al. [3])
	UZ220909-2978-1	Human synovial fluid	Own isolate
S. agalactiae	CCUG49100	Human synovial fluid	CCUG
E. faecalis	CCUG35128	PJI knee	CCUG
C. acnes	CCUG48138	Human synovial fluid	CCUG
E. coli	UZ220830-1777	Human synovial fluid	Own isolate
	UZ220829-0412	Human synovial fluid	Own isolate
P. aeruginosa	UZ230201-3568-1	Artrex anker	Own isolate
	UZ230406-3644-1	Chronic osteomyelitis	Own isolate
C. parapsilosis	UZ220929-2760-1	Human synovial fluid	Own isolate
C. albicans	UZ221012-3305-1	Human synovial fluid	Own isolate

Table S2. Antimicrobials selected to determine antimicrobial susceptibility.

Microorganism	Antibiotics
Staphylococcus spp.	Oxacillin, rifampicin, doxycycline, cefazolin
Streptococcus spp.	Amoxicillin, doxycycline, ceftriaxone, cefazolin
Enterococcus spp.	Amoxicillin, doxycycline, vancomycin, cefazolin
Gram negatives	Ciprofloxacin, cefazolin
Cutibacterium spp.	Amoxicillin, rifampicin, doxycycline, ceftriaxone, cefazolin
Candida spp.	Fluconazole, cefazolin

Table S3. Maximum antimicrobial concentration tested for the susceptibility determinations.

**Antimicrobial agent**	Oxacillin	Rifampicin	Doxycycline	Cefazolin	Amoxicillin	Ceftriaxone	Vancomycin	Ciprofloxacin	Fluconazole
**Concentration (µg/mL)**	1024	32	1024	1024	1024	1024	1024	1024	512

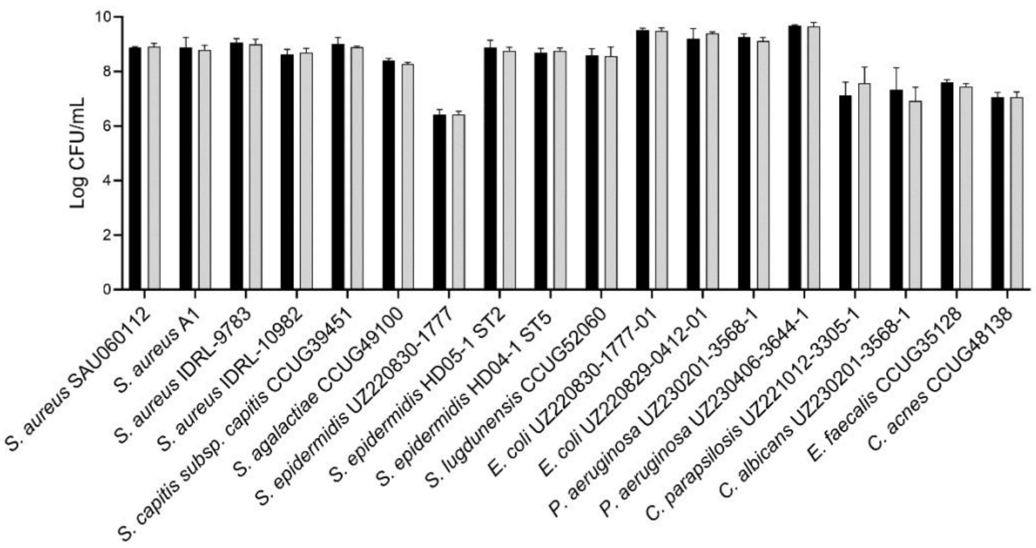

Figure S1. Log CFU/mL values of 18 clinical PJI isolates after 24 h of incubation in MHB under aerobic conditions (except for *C. acnes*; 48 h of incubation under anaerobic conditions), obtained after conventional biofilm disruption (black bars) and trypsin treatment (grey bars). Data shown are mean values of biological replicates, error bars represent standard deviation (n=3). No significant differences were observed.

Protocol for preparation of 100 mL SSF2 medium

The solid components listed in Table 1 need to be dissolved in the different liquid components listed in Table 2. First weigh the bovine serum albumin (BSA) (Merck Life Science, Darmstadt, Germany) and dissolve in the required volume of MilliQ (MQ) water. Next add all desired volumes of the stock solutions, except for fibrinogen (Merck Life Science, Darmstadt, Germany), and filter sterilize (0.22 µm) the whole. Subsequently, add the UV-sterilized sodium hyaluronate (1.20-180 MDa) (Lifecore Biomedical, Menlo Park, California, USA) and mix the whole for 30 min, using a shaker (250 rpm) at 37°C. Finally, add the required volume of the fibrinogen stock. Preparation of the medium, starting from the stock solutions, including the shaking, takes approximately 1h. Please note that for the preparation of SSF1, the same protocol can be followed, without addition of the fibrinogen stock and with 4 mL of additional MQ water instead.

Table 1. Solid components SSF2 medium

Component	Weight
BSA	2.5 g
Sodium Hyaluronate	0.3 g

Table 2. Liquid components SSF2 medium

Component	Volume for 100 mL
MQ	74.37 mL
M9 salts	10 mL
Glucose	2 mL
$MgSO_4$	100 µL
$CaCl_2$	30 µL
Trace elements	1 mL
Tyrosine	5 mL
Glutamic acid	2 mL
Amino acids	1 mL
Nicotinic acid	100 µL
Thiamine	100 µL
Calcium pantothenate	100 µL
Biotin	100 µL
Citric acid	100 µL
Fibrinogen	4 mL

Preparation of the stock solutions

Trace elements

Prepare the stock solutions required for the trace element solution with the concentrations listed in Table 4 and filter sterilize (0.22 µm). Subsequently, dissolve 5 g of Na_2-EDTA (Merck Life Science, Darmstadt, Germany) in 800 mL MQ water and adjust the pH to 7.5. Finally, combine the required volume of the stock solutions with the required amount of the solid compounds as listed in Table 4 to obtain the final trace element solution.

Table 3. Trace elements for the solution

Trace element	Amount
$FeCl_3$ [a]	498 mg
$ZnCl_2$ [b]	84 mg
$CuCl_2.2H_2O$ [a] (0.1 M)	765 µL
$CoCl_2.6H_2O$ [a] (0.2 M)	210 µL
H_3BO_3 [c] (0.1 M)	1.6 mL
$MnCl_2.4H_2O$ [a] (1 M)	8.1 µL

[a] Merck Life Science, Darmstadt, Germany
[b] Honeywell, Charlotte, USA
[c] Janssen Chimica, Beerse, Belgium

Amino acids

Dissolve all required amounts of amino acids listed in Table 5 in 1L of MQ water and filter sterilize (0.22 µm) the whole.

Table 4. Amino acids

Amino acid	Weight
L-Alanine [a]	5 g
L-Arginine [b]	0.89 g
L-Asparagine [a]	1.2 g
L-Aspartic acid [a]	0.88 g
L-Cysteine [a]	2 g
L-Glutamine [a]	7.6 g
L-Glycine [a]	2.9 g
L-Histidine [a]	1.8 g
L-Isoleucine [a]	1.3 g
L-Leucine [a]	2.7 g
L-Lysine [a]	3.5 g
L-Methionine [a]	0.43 g
L-Phenylalanine [c]	1.6 g
L-Proline [d]	2.8 g
L-Serine [a]	2 g

L-Threonine [c]	1.9 g
L-Tryptophan [a]	1.4 g
L-Valine [a]	2.7 g

[a] Merck Life Science, Darmstadt, Germany
[b] Acros Organics, Geel, Belgium
[c] Honeywell, Charlotte, USA
[d] Fisher Scientific, Waltham, USA

M9 salts

For a volume of 1L M9 salts, prepare by dissolving the salts listed in Table 6 in 800 mL MQ water, adjusting the pH to 7.2 and diluting with MQ water to a final volume of 1 L. Autoclave the whole at 121°C for 20 minutes.

Table 5. M9 salts

Salt	Weight
$Na_2HPO_4.2H_2O$ [a]	75.2 g
KH_2PO_4 [b]	30 g
NaCl [c]	5 g
NH_4Cl [d]	5 g

[a] Janssen Chimica, Beerse, Belgium
[b] Honeywell, Charlotte, USA
[c] Chemlab Analytical, Zedelgem, Belgium
[d] Fisher Scientific, Waltham, USA

Remaining stock solutions

Weigh the amounts listed in Table 7, dissolve in the corresponding volume MQ water and filter sterilize (0.22 µm). Except for fibrinogen, which needs to be dissolved in PS and cannot be filter sterilized (0.22 µm) due to its viscosity. It can neither be UV-sterilized, as this can compromise its stability. For this reason, always check its sterility by making a new batch.

Table 6. Remaining stock solutions

Component	Weight	Volume
Glucose [a]	2.5 g	50 mL
$MgSO_4$ [a]	1.233 g	5 mL
$CaCl_2$ [a]	725 mg	5 mL
L-Tyrosine [a]	28 mg	100 mL
L-Glutamic acid [a]	96 mg	50 mL
Nicotinic acid [a]	10 mg	5 mL
Thiamine [a]	10 mg	5 mL
Calcium pantothenate [b]	10 mg	5 mL
Biotin [a]	0.5 mg	5 mL
Citric acid [a]	120 mg	5 mL
Fibrinogen [a]	25 mg	10 mL

[a] Merck Life Science, Darmstadt, Germany
[b] Fisher Scientific, Waltham, USA

<u>Recommendations regarding shelf life and storage</u>

All stock solutions can be frozen (at -20°C) for at least a year (except for the fibrinogen stock, which is always freshly prepared). The prepared SSF solutions can be stored frozen (at -20 °C) for maximum 6 months or cooled (at 4-7°C) for maximum 2 weeks.

References supplementary data

[1] Y. Xu *et al.*, “In vivo gene expression in a Staphylococcus aureus prosthetic joint infection characterized by RNA sequencing and metabolomics: A pilot study,” *BMC Microbiol.*, vol. 16, no. 1, May 2016, doi: 10.1186/s12866-016-0695-6.

[2] T. Le Masters *et al.*, “Comparative Transcriptomic Analysis of Staphylococcus aureus Associated with Periprosthetic Joint Infection under in Vivo and in Vitro Conditions,” *J. Mol. Diagnostics*, vol. 23, no. 8, pp. 986–999, Aug. 2021, doi: 10.1016/j.jmoldx.2021.05.011.

[3] A. Both *et al.*, “Distinct clonal lineages and within-host diversification shape invasive staphylococcus epidermidis populations,” *PLoS Pathog.*, vol. 17, no. 2, Feb. 2021, doi: 10.1371/JOURNAL.PPAT.1009304.
